# Progression to Decompensation of Severe Fibrosis Compared to Cirrhosis in MASLD: A Systematic Review and Meta‐Analysis

**DOI:** 10.1111/liv.70511

**Published:** 2026-01-17

**Authors:** Rachael Barrett, Annie Archer, Jennifer Cathcart, Kushala Abeysekera, John F. Dillon, Paul N. Brennan

**Affiliations:** ^1^ School of Medicine University of Dundee, Ninewells Hospital and Medical School Dundee UK; ^2^ Bristol Medical School Bristol UK

## Abstract

**Background & Aims:**

Metabolic associated steatotic liver disease (MASLD) is increasing in prevalence worldwide. Clinical practice is focused on identifying those with cirrhosis and monitoring for complications such as varices and hepatocellular carcinoma (HCC). Non‐invasive tests of fibrosis differentiate between F3 and F4 fibrosis poorly. People with F3 fibrosis may progress and develop decompensated liver disease. The aim of this review is to examine the progression to decompensated liver disease in patients with F3 fibrosis compared to those with F4 fibrosis.

**Methods:**

Searches were carried out in four databases; articles were screened by two independent reviewers against pre‐specified inclusion and exclusion criteria.

**Results:**

Twenty‐nine studies were included in the review: 12 with paired liver biopsies, 2 progression to cirrhosis, 13 progression to decompensation, 2 portal hypertension in F3 fibrosis and 13 on HCC in F3 fibrosis. Rates of progression on paired biopsies were 16%–30% over varied follow‐up. Varices were found in 16% of patients with F3 fibrosis and rates of non‐cirrhotic HCC varied from 37%–75%. Pooled univariate HR for F3 progression and F4 progression to major adverse liver outcomes (MALO) were 8.15 (95% CI 3.42–19.43) and 38.16 (95% CI 11.58–125.76), respectively.

**Conclusions:**

Progression to cirrhosis and decompensation events occurs in a significant proportion of patients with F3 fibrosis in MASLD. There is evidence of portal hypertension and HCC developing in F3 MASLD. Further work to identify risk groups, including those at risk of rapid progression to guide future clinical management is urgently required given the prognostic inflection of decompensated disease.

## Key Points

- Progression to cirrhosis and decompensation occurs in a significant proportion of patients with MASLD and F3 fibrosis.
- There is a risk of developing HCC in F3 MASLD, particularly in men.
- Clinically significant portal hypertension can develop in F3 MASLD.
- It takes roughly double the time for patients with F3 fibrosis to develop a decompensating event compared with those with F4 fibrosis.

**Abbreviations**
APRI: AST to Platelet Ratio Index
CSPH: clinically significant portal hypertension
CTP: Child‐Turcotte‐Pugh
dACLD: decompensated advanced chronic liver disease
ELF: enhanced liver fibrosis test
FIB‐4: Fibrosis‐4 Index
GOV: gastro‐oesophageal varices
HCC: hepatocellular carcinoma
INR: international normalised ratio
LSM: liver stiffness measurement
LSM‐VCTE: liver stiffness measurement–vibration controlled transient elastography
MASH: metabolic dysfunction‐associated steatohepatitis
MASLD: metabolic dysfunction‐associated steatotic liver disease
MetALD: metabolic dysfunction and alcohol related liver disease
MRE: Magnetic Resonance Elastography
NAFLD: non‐alcoholic fatty liver disease
NASH: non‐alcoholic steatohepatitis
NIH: National Institute of Health
OGD: oesophago‐gastroduodenoscopy
PICO: population intervention comparison outcomes
RCT: Randomised Controlled Trial
SBP: spontaneous bacterial peritonitis
T2DM: type 2 diabetes mellitus

## 1 Introduction

Metabolic associated steatotic liver disease (MASLD), previously termed non‐alcoholic fatty liver disease (NAFLD), is increasing in prevalence worldwide alongside increasing incidence of obesity [1] and type 2 diabetes mellitus [2]. The new consensus nomenclature acknowledges the existence of MASLD in those who consume greater amounts of alcohol per week (140–350 g/week for females and 210–420 g/week for men) which is termed MetALD [3, 4]. This categorisation better reflects real‐world evidence, where metabolic risk factors co‐exist with alcohol, viral hepatitis and autoimmune conditions. The prevalence of the MASLD spectrum is now estimated to be around 38% [5], whilst the majority of these patients have simple steatosis; an estimated 1 in 40 people in the United States general population have steatohepatitis based on the latest studies [6].

Current practice focuses on identifying those with at least F3—F4 fibrosis using non‐invasive fibrosis scores and serum markers such as the Enhanced Liver Fibrosis (ELF) score [7]. The cutoff for ELF presently used, 9.8, has a high specificity of 98% for identification of moderate fibrosis but a lower sensitivity of 69% [8]. Current screening models are based on sequential testing with non‐invasive scores such as Fibrosis‐4 Index (FIB‐4) and then proceeding either directly to liver stiffness measurement by vibration‐controlled transient elastography (LSM‐VCTE) or further risk stratifying with ELF test prior to proceeding to LSM [9, 10]. It has been shown that the strategy of FIB‐4 followed by ELF in indeterminate cases improves diagnostic accuracy and results in manageable numbers of referrals whilst keeping false negatives to less than 10% [10]. Current hepatology practice for follow up recommends screening for varices and HCC surveillance in patients with cirrhosis, not in those with severe fibrosis. However, current NITS differentiate F3 and F4 poorly with considerable overlap and even liver biopsy can underdiagnose cirrhosis. It would be beneficial to know what the complication rates are in those graded F3 to ascertain the benefit of including them in screening and surveillance cohorts, reducing the need for differential diagnosis between F3 and F4.

Liver related mortality is increased in patients with MASLD and F3 (marked bridging, fibrosis with expansion of most portal zones) or F4 (cirrhosis) [11]. Multi‐national cohort modelling has projected MASLD and decompensated liver disease related mortality to double between 2016 and 2030 [12], with some guidelines now suggesting individualised risk assessment for people with MASLD and F3 fibrosis [13]. Fibrosis stage is the main predictor of liver associated events [14] with liver related mortality increased in patients with MASLD and F3 or F4 fibrosis [11]. Historically, MASLD fibrosis progression was considered slow, with Singh et al.'s meta‐analysis of paired biopsy NAFLD cohorts reporting progression by one stage of fibrosis occurring over 14.3 years for patients with MASLD and 7 years for those with metabolic dysfunction‐associated steatohepatitis (MASH) [15]. The authors found there were two subsets of patients: ‘rapid progressors’ and ‘slow progressors’ but did not report clinical outcomes. Given the risk in progression of fibrosis stage, its association with adverse outcomes and significant disease burden in younger adults, it could be argued that those with F3 fibrosis warrant ongoing monitoring in hepatology services. Current practice is focused on identifying those with cirrhosis without clarity on how best to follow‐up intermediate results.

The aim of this review was to examine the progression to decompensated liver disease in those patients with MASLD and F3 fibrosis compared with those patients with F4 fibrosis. This understanding may guide timely population‐based screening strategies, prognostic stratification, and suitable follow‐up for patients at risk of advanced liver fibrosis. We will use the terms MASLD/MASH in this paper as interchangeable with NAFLD/NASH for those studies reporting results under the previous nomenclature [16].

## 2 Methods

### 2.1 Criteria for Considering Studies for This Review

The population of interest for inclusion were all adults (> 18 years) with F3 liver fibrosis from NAFLD or NASH aetiology; we included MAFLD and MASLD in this terminology. The comparator group was patients with F4 liver fibrosis. There was no specific intervention as we were interested in follow‐up over time and the outcome of interest was decompensation of liver cirrhosis. Observational and randomised controlled study designs were accepted. Prior to searches being run, this systematic review was registered in PROSPERO, CRD42023463365. A study protocol was pre‐specified and is available on request. Studies not in the English language, studies without complete datasets, reviews and systematic reviews were excluded. Search terms were developed in conjunction with support from the academic librarian team. PICO question can be found in Table S1.

The specific questions for this review are as follows:

1. Do patients with F3 fibrosis secondary to MASLD carry significant risk of progression to decompensated liver disease?
2. What is the rate at which F3 fibrosis progresses to F4 fibrosis in paired liver biopsies and what are the rates of regression of F3 fibrosis to F0‐2?
3. What is the incidence of HCC development in patients with F3 MASLD/MASH patients?
4. What is the evidence of development of portal hypertension in patients with MASLD and F3 fibrosis?

### 2.2 Search Methods for Identification of Studies

Databases searches were run on 30.10.2023, 31.10.2023 and 02.11.2023 in Pubmed, The Cochrane Library, Embase and Scopus; with no restrictions placed on publication date. During the data extraction any papers cited in the discussion of included papers that met our inclusion criteria were added. In the event of abstract only titles, we searched for a full text version, and if found, replaced the abstract with full text version. If no full text version was available and the abstract had sufficient information to fulfil the inclusion criteria the abstract was included. Rayyan was used to assist in duplication removal and for screening [17]. Screening was carried out by two independent blinded researchers (R.B. and J.C.), with any disagreements resolved following discussion with the study team. The searches used can be found in Tables S2–S5.

### 2.3 Assessment of Risk of Bias

Bias risk assessment was carried out by R.B. and J.C. using the NIH Quality Assessment Tool for Observational Cohort and Cross‐Sectional Studies.

### 2.4 Data Extraction and Synthesis

Data extraction was performed by one researcher (R.B.) using a pre‐specified proforma in Excel. The outcomes for which data were sought were progression to decompensated liver disease (encephalopathy, ascites, variceal bleeding), development of HCC, diagnosis of oesophageal or gastric varices in F3 MASLD, progression of F3 MASLD on paired biopsy studies and regression of F3 fibrosis on paired biopsy studies. Other data items sought were study funder, conflicts of interest, aim of paper, inclusion and exclusion criteria, setting, methods of recruitment, sample size, study design, measurement of fibrosis, intervention (if applicable), follow‐up period, demographics (age, sex, ethnicity, severity of illness), co‐morbidities, imputation of missing results, statistical methods and conclusions of study authors. Included titles were visually grouped and then tabulated under the objectives of the review, and narrative synthesis performed. Meta‐analysis of univariable hazard ratio effect estimates for F3 and F4 progression to decompensated liver disease outcomes was attempted using the restricted maximum likelihood (REML) model. Multivariable pooled hazard ratio estimates were not reported due to differences in covariates used between individual studies. Analysis was performed on Stata 18.0 using the *meta* function.

## 3 Results

### 3.1 Included Studies

**FIGURE 1:**
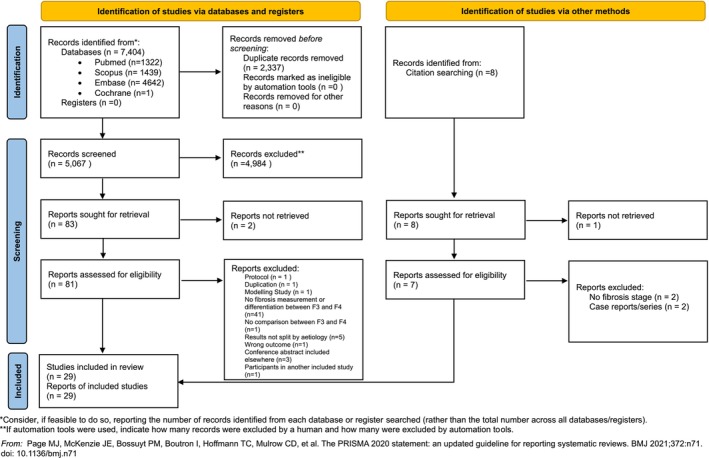
PRISMA flow diagram.

The searches yielded 7404 results, 2337 duplicates were removed with the assistance of Rayyan [17] with the automatic de‐duplication threshold set to 98% and the rest resolved manually. This left 5067 articles for screening. 4894 results were excluded at title and abstract screening stage with 83 progressing to full text review. Fifty‐seven were excluded at full text review and three additional studies included from the references of other papers giving 29 studies (5 abstracts and 24 full text articles) for inclusion, see Figure 1.

### 3.2 Demographics

Demographics of the included studies can be found in Table S6.

### 3.3 Assessment of Fibrosis

The majority of studies (27) assessed fibrosis with either liver biopsy or histology from resected liver tissue, 1 by LSM‐VCTE and 1 by MRE. Three studies used LSM in addition to histology.

### 3.4 Risk of Bias

Bias assessment was carried out with the NIH tool for cohort studies, see Supporting Information for ROBVIS tool [18]. We graded 19 studies as good, 9 as fair and 1 as poor. All studies were retained for data synthesis. We aimed to minimise risk of publication bias by including conference abstracts in the synthesis.

### 3.5 Data Synthesis

We grouped studies to address the pre‐specified objectives and undertook a narrative synthesis of the data.

#### 3.5.1 Progression on Paired Biopsies

Eight studies were identified reporting on progression of F3 fibrosis in paired liver biopsies [19, 20, 21, 22, 23, 24, 25, 26]. Follow‐up in these studies was at least 1 year between liver biopsies and up to 7 years. Most studies reported progression rates of 16%–25% [19, 20, 21, 22, 23, 25] with two studies reporting progression in 50% of participants, both of these studies had small numbers of participants (2 and 4) with F3 fibrosis [24, 26]. There was overlap in the participants in three of these studies, which included participants from the simtuzumab and selonsertib studies [19, 20, 21], which were terminated early due to lack of efficacy of the investigational medicinal product.

#### 3.5.2 Regression on Paired Biopsies

We found five studies reporting regression in paired liver biopsies [21, 22, 23, 26, 27]. Four papers reported rates of regression between 21% and 34% overall [21, 22, 23, 26], with one paper reporting 100% regression but this paper had only one participant with F3 fibrosis [27]. Sanyal et al. [21] found 21% of patients with bridging fibrosis had at least 1 stage reduction in fibrosis, and 8.6% of those with cirrhosis at baseline had regression on repeat biopsy. In Huang et al.'s study of those with T2DM who had F3 fibrosis at baseline (*n* = 49), 21 had regression on repeat biopsy; 1 (1.4%) regressing to F0, 8 (12%) to F1 and 12 (17%) to F2. In those without T2DM, out of 46 patients with F3 fibrosis 26% regressed at follow‐up, 6 patients to F1 and 6 patients to F2 [22].

#### 3.5.3 Progression to Cirrhosis

Most studies reported on progression in paired biopsies or progression to decompensation, but some studies commented on progression to cirrhosis. Gawrieh et al. [28] looked at participants with biopsy confirmed MASLD and defined progression to cirrhosis as LSM ≥ 14.9 kPa; progression was found in 24% with F3 and 30% with F4 fibrosis by histology at baseline, which was statistically significant versus lower grades of fibrosis. In Loomba et al.'s paired biopsy study, risk of progression was greater with higher LSM; the optimal threshold was ≥ 16.6 kPa. Baseline progression to cirrhosis occurred in 31.1% of participants with LSM ≥ 16.6kPa compared with 9.1% with a LSM < 16.6 kPa [20].

#### 3.5.4 Progression to Decompensation

We found 13 papers examining progression to decompensation, HRs if reported are summarised in Table 1 [14, 19, 21, 26, 29, 30, 31, 32, 33, 34, 35, 36, 37] and event rates in Table S8. Varying event rates for F3 and F4 fibrosis were reported. Younossi et al. [19] reported 16.7% of patients with bridging fibrosis progressed, with 7% of these developing a clinical event (1.2% of the total cohort). In those with cirrhosis at baseline, however, 7% experienced decompensation or other liver related event during median follow‐up of 16 months [21]. Hagstrom et al. reported progression in 12.1% of participants with F3 at baseline and 45% of those with F4 at baseline, including progression to cirrhosis and decompensation, similar to Angulo et al. who reported clinical events in 13.7% of patients with F3 fibrosis at baseline and 23.5% of those with F4 fibrosis at baseline [14, 29]. Vilar‐Gomez et al. [32] reported their results by Child‐Turcotte‐Pugh (CTP) score, finding hepatic decompensation developed in 19% of those with F3 fibrosis, and 59% and 85% of those with F4 fibrosis Child‐Turcotte‐Pugh score (CTP) A5 and A6, respectively.

**TABLE 1:** Hazard ratios for development of decompensated liver disease or severe liver disease (95% confidence interval).

Study	Number of participants	F3 fibrosis	F4 fibrosis	Follow‐up period	Definition in paper
**Decompensated liver disease**					
Sanyal 2021	1773	HR 18.7 (4.8–73.1)	HR 36.1 (8.9–146.3)	Mean: 4.0 years	New onset hepatic decompensation = clinically apparent ascites, overt encephalopathy or variceal haemorrhage
Kobayashi	405	HR 4.81 (0.4–54.3)	HR 24.84 (4.51–137.0)	Mean: 6.1 years	Decompensated cirrhosis = at least one of the following findings: gastroesophageal variceal haemorrhage, ascites, hepatic encephalopathy, or jaundice
**Liver related event or severe liver disease**					
Hagstrom	646	HR 14.3 (7.9–25.8)	HR 104.5 (57.2–191.1)	Mean: 19.9 years	Severe Liver Disease = ICD code for liver failure, cirrhosis, HCC or decompensated liver disease Decompensated liver disease in turn defined as ICD‐code: oesophageal varices (bleeding or not bleeding), ascites or hepatic encephalopathy
Fujii	1398	HR 28.7 (3.35–246.0)	HR 135.8 (15.0–1231.0)	Median: 4.6 years	Liver‐related events = GOV/bleeding, HCC or decompensated cirrhosis
Angulo	619	HR 13.4 (4.2–42.6)	HR 52.9 (13.3–210.2)	Median: 12.6 years	Liver related event: GOV or bleeding, ascites, portosystemic encephalopathy, SBP, HCC hepatopulmonary syndrome, hepatorenal syndrome
Hirose	233	HR 4.1 (0.8–21.1)	HR 29.2 (5.8–148.6)	Mean: 12.1 years	Liver‐related events = HCC, variceal bleeding, encephalopathy, risk of comorbidities
Lam	450	HR 2.5 (1.1–5.9)	HR 6.3 (3.2–12.1)	Median: 3.2 years	Liver‐related events but not defined

Kobayashi et al. assessed patients with MASLD who had at least two Magnetic Resonance Elastography (MRE) results finding 3 out of 62 (cumulative probability 4.8%) of those with F3 at baseline developed decompensated cirrhosis versus 6 out of 47 of those with F4 fibrosis at baseline (cumulative probability 12.8%) after a mean of 72.6 months follow‐up [35].

Hagstrom et al. [29] reported the time until 10% of patients developed severe liver disease was 6 years in F3 fibrosis. People with F4 fibrosis at baseline decompensated in roughly half the time of F3, with time until 10% having decompensation being 11.8 years and 5.6 years for F3 and F4 fibrosis, respectively [29]. In another study all participants with F4 fibrosis at baseline had an event by 14 years and for F3 fibrosis only 3 participants (*n* = 51) reached 20 years without a liver‐related event [14].

#### 3.5.5 Portal Hypertension in F3 MASLD

We found two papers reporting specifically on development of gastro‐oesophageal varices (GOV). Nakamura et al. looked at participants with biopsy proven MASH who had an OGD at the time of liver biopsy, including 72 participants with 34 having GOV at OGD. Varices were found in 4/25 (16%) of people with bridging fibrosis and 30/47 (63.8%) of those with cirrhosis [38]. Sanyal et al. reported event rates of variceal bleeding in their study as F3 1/362 0.06 per 100 person years, F4 5/163 0.7 per 100 person years [33].

#### 3.5.6 Hepatocellular Carcinoma

Six studies were identified reporting rates of HCC development in patients with F3 fibrosis at baseline. One paper found HCC was numerically higher in F3 than F4 fibrosis (0.34 per 100 person‐years versus 0.14 events per 100 person‐years, respectively) [33]. Others found more HCC developing in participants with F4 at baseline but did find HCC in F3 MASLD, see Table 2 and Table S9 [32, 34, 35, 39]. One study reported that only stage 4 fibrosis had a significantly increased risk of HCC developing compared to stage 0–1 fibrosis (HR 16.9, 95% CI 1.95–147.40) [37].

**TABLE 2:** Incidence rates and hazard ratios for developing HCC.

Study	Number of participants	F3 fibrosis	F4 fibrosis
Kobayashi	405	HR 33.0 (95% CI 3.8–288.9)^a^	HR 45.3 (95% CI 6.2–333.0)^a^
Vilar‐Gomez	458	Annualised incidence rate 0.2 (95% CI: 0.02–0.9)	Annualised incidence rate CTP A5: 1.8 (95% CI: 1.1–2.7) CTP A6: 4.7 (95% CI: 3.0–7.5)
Davitkov	13 629 MASLD 42 HCC	Incidence: 0.2/100 patient years (95% CI: 0.05–0.51) IRR 4.81 (95% CI: 1.19–8.2)^b^	Incidence: 1.02/100 patient years (95% CI: 0.68–1.46) IRR of 1.74 (95% CI 0.69‐5.88)^c^
Sanyal	475	Incidence: 0.34 per 100 person years HR: 9.3 (95% CI: 1.4–61.8)^d^	Incidence: 0.14 events per 100 person years HR 4.9 (95% CI: 0.4–63.2)^d^

^a^ Compared to F0 fibrosis.

^b^ LSM‐VCTE: 9.5–12.4 kPa, IRR relative to < 9.5 kPa group.

^c^ LSM‐VCTE: ≥ 14.5 kPa, IRR relative to 9.5–12.4 kPa group.

^d^ Compared to F0‐2 fibrosis.

Six studies reported on fibrosis stage at the time of HCC diagnosis. Kodama et al. found 22.1% and 44.2% had F3 and F4 fibrosis, respectively, in 104 patients diagnosed with MASLD‐HCC, with men developing HCC at lower stages of fibrosis than women [40]. Similarly, Yasui et al. described that females with HCC were more likely to have cirrhosis, and males developed HCC at lower fibrosis stages; F3 fibrosis was present in 18 patients (21%) and in 44 patients with F4 fibrosis (51%) [41]. Kawada et al. found 8 out of 807 patients undergoing liver resection for MASLD HCC were diagnosed with MASH. Of these, 75% had non‐cirrhotic background liver [42]. Mohammad et al. found that 36 out of 83 patients had non‐cirrhotic liver (F0 55.9%, F1 17.6%, F2 8.8%, F3 17.6%) in MASLD‐HCC, additionally finding the non‐cirrhotic group were more likely to present with a single and larger nodule [43]. Bengtsson et al. looked at MASLD‐HCC deeming in 225 patients; 83 (37%) did not have cirrhosis and were more likely to be older, less likely to have T2DM and have a larger total tumour size. Fibrosis stage was ascertained in 35 of these patients as follows: F0 = 1, F1 = 13, F2 = 16, F3 = 5 [44]. Pinyopornpanish et al. [45] reported the following rates of fibrosis in patients with non‐cirrhotic MASLD who were diagnosed with HCC: F0 23 people (39.7%), F1 17 people (29.3%), F2 7 (12%) and F3 11 people (19%).

### 3.6 Meta‐Analysis

**FIGURE 2:**
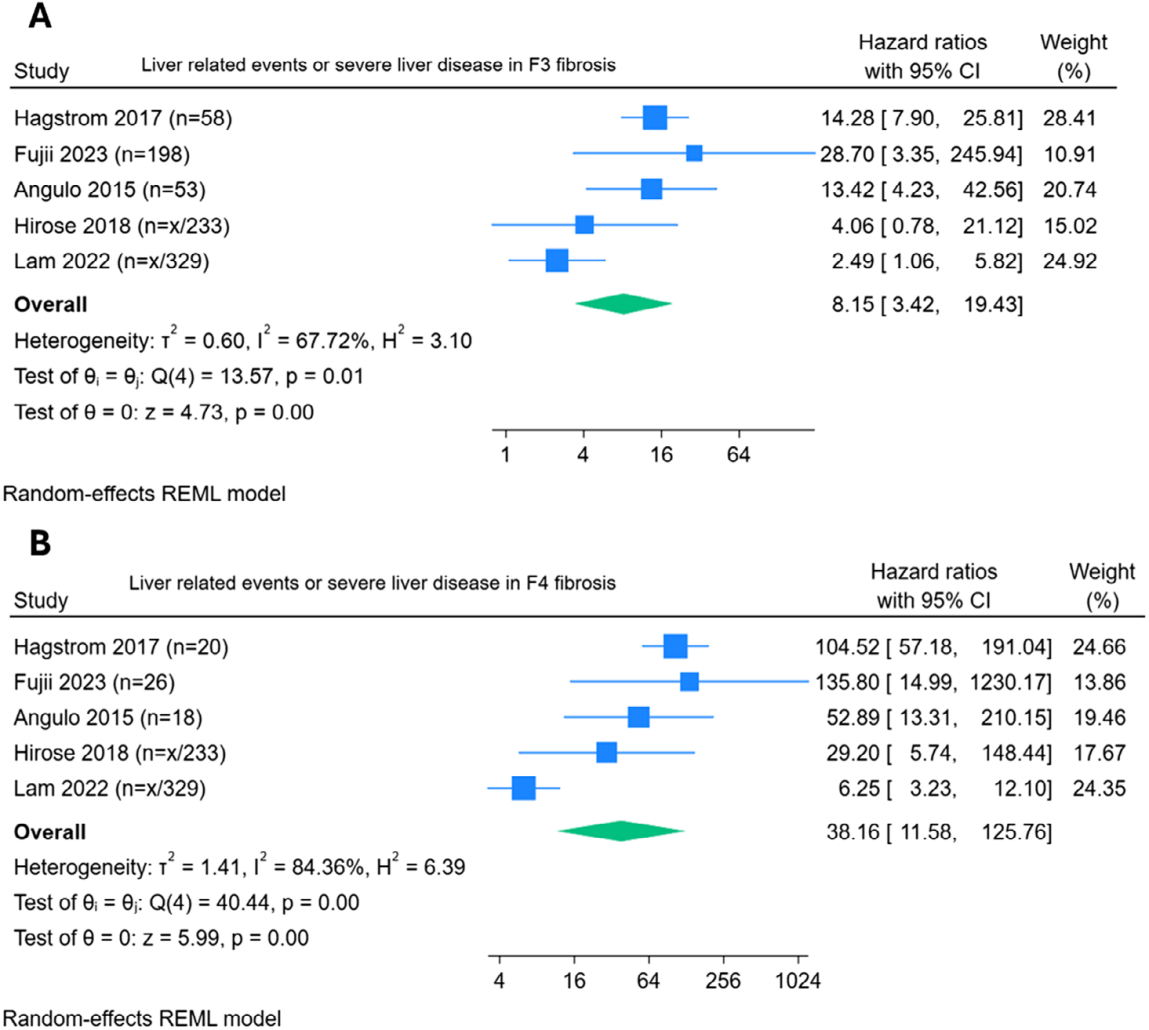
Forrest plots, liver related events in F3 (A) and F4 (B) fibrosis.

Five studies provided univariable hazard ratios of major adverse liver outcomes (MALO) [14, 29, 30, 36, 37] amongst patients with F3 fibrosis, with a pooled HR 8.15 (95% CI 3.42–19.43; *I* ^2^ 67.7%, *τ* ^2^ 0.9). The pooled HR for patients with F4 fibrosis developing major adverse liver outcomes (MALO) was over 4 times greater HR 38.16 (95% CI 11.58–125.76; *I* ^2^ 84.4%, *τ* ^2^ 1.4). See Figure 2. A sensitivity analysis removing those studies rated ‘fair’ in risk of bias assessment can be found in Figures S1 and S2. Univariable meta‐regression was performed for sex, type 2 diabetes and location which can be found in Table S7.

## 4 Discussion

In this systematic review we screened 5067 journal articles and included 29 in the data synthesis presenting relevant results to allow better understanding of progression to cirrhosis, decompensation and development of hepatocellular carcinoma; in patients with F3 fibrosis compared with F4 fibrosis secondary to MASLD. We found that in paired biopsy studies between 16.7% and 50% progressed to F4 fibrosis over mean follow‐up ranging from 3.3 years to 6.3 years. Most studies suggested that progression to decompensation was more likely and quicker in F4 fibrosis versus F3, but the progression rates in F3 were still compelling. The results presented with regards to HCC show that a significant proportion of those diagnosed with MASLD‐HCC do not have cirrhosis in the background liver tissue. Furthermore, we found evidence of portal hypertension developing in F3 MASLD. It is important to acknowledge that the definition of F3 and F4 is based on semi‐quantitative subjective measures that are known to be subject to sampling and observer error. Furthermore, the non‐invasive tests of fibrosis have been validated against this suboptimal standard. This is why it is important to validate these grading systems against clinical outcomes.

We found that progression to cirrhosis occurred in 16% [19] and 22% [21] of patients with F3 fibrosis in prospective studies. These findings, however, were reported from randomised controlled trials of therapeutic agents for MASLD which showed no benefit and may not be fully representative of the natural course of the disease. Most patients with disease progression (93% and 87.5%) progressed histologically, with the remainder deemed to progress to cirrhosis based on clinical assessment. This suggests that simply following patients in terms of clinical assessment may not identify the majority of those who have progressed to cirrhosis and subsequently would benefit from variceal and HCC screening. Conversely, one included paper showed the same rates of decompensation for F3 and F4 fibrosis [26] and several others had overlapping HRs for decompensation in the two fibrosis stages [14, 33, 35]. An observational study of 1893 patients with biopsy proven MASLD found that patients meeting the diagnostic criteria for MASH were more likely to progress to cirrhosis than those with MASLD [46]. Decompensation was not consistently defined across the included studies, with some including jaundice in the definition. This is comparable to other literature, with a systematic review finding 50 out of 88 studies with a definition of decompensation included jaundice in their criteria [47]. The identification of patients who are going to progress presents a conundrum for hepatologists who will need to balance the opportunity costs of following up all patients with advanced fibrosis.

In the included studies, many factors were identified as predictors of progression including T2DM [22], INR, platelets, ELF, APRI, NAFLD score and FIB‐4, with the optimum ELF to predict progression to cirrhosis ≥ 9.76 kPa (sensitivity 77%, specificity 66%) [21]. ELF cutoff of ≥ 11.3 was found to have a specificity of up to 94% and positive predictive value of up to 48% for prediction of progression of patients with MASH and F3 fibrosis at baseline [19]. Hagstrom et al. showed a pronounced difference in time until 10% of people developed severe liver disease (liver failure, cirrhosis, HCC or decompensated liver disease) between F2 and F3 fibrosis (19.4 years and 6.0 years, respectively) [29], and it is in fact this difference which may convince the hepatology community that identifying patients with F3 fibrosis and monitoring for progression is necessary. Evidence was also presented that lower baseline ELF and lower NAFLD fibrosis score were predictive of regression on fibrosis [21]. One paper, with biopsy assessment of fibrosis stage, found the optimal baseline LSM by VCTE to predict progression to cirrhosis was ≥ 16.6 kPa, at which progression to cirrhosis occurred in 31.1% of patients compared with 9.1% who had LSM < 16.6 kPa [20]. Duseja et al. classified LSM > 13 kPa as compensated cirrhosis and found 36% of patients in this category had decompensating events versus only 1.2% in those with LSM 8–13 kPa [34]. Further work around these risk factors and optimal thresholds for non‐invasive assessment of liver fibrosis would guide development of care pathways for identification and follow‐up of at‐risk populations.

Portal hypertension was detected in F3 MASLD, with varices found in 16% of patients with F3 fibrosis compared with 63.8% of those with F4 fibrosis [38]. The rate of variceal bleeding in those with F3 fibrosis was noted to be much lower than F4 fibrosis, reported by Sanyal et al. [33]. Interestingly, the authors did not find any variceal bleeding in patients with F0‐2 fibrosis, consistent with the hypothesis that there is a difference in risk of decompensating event in F3 versus F0‐2 fibrosis.

We identified contradictory evidence of the development of HCC in MASLD. Firstly, one study found there were a numerically increased number of HCC in F3 fibrosis than F4 fibrosis [33], but this represents relative differences in competing risk, especially for those with F4 disease, who are more likely to succumb to decompensated advanced chronic liver disease (dACLD). Others looked at the fibrosis stages in patients diagnosed with HCC and found 22.1% of these patients had F3 fibrosis and 44% of patients had F4 fibrosis [40]. If this was corroborated in a trial with larger patient numbers, this may have implications for HCC screening programmes. We found rates of non‐cirrhotic HCC varying from 37% to 75% of those diagnosed with HCC in MASLD. The higher rates of 75% were found in patients with MASH, and this may indicate a subgroup of patients who require closer monitoring for the development of HCC. A previous study with 4236‐person year follow‐up did not observe any HCC in patients with MASH and F3 fibrosis [48] but patients were censored at diagnosis of F4 fibrosis or clinical portal hypertension and, therefore, cases could've been missed from the analysis if the patient progressed to cirrhosis prior to developing HCC. This is an inherent risk of non‐competing risk analysis, and further prospective work using a competing risk analysis may help to clarify rates of development or MASLD in F3 fibrosis. Males were found to develop more HCC at lower stages of fibrosis than females in two included studies [40, 41] which opens debate to sex stratified thresholds for HCC screening as proposed in a recently developed risk assessment score [49]. Overall, HCC surveillance in patients with non‐cirrhotic MASLD is not recommended in recent British Society of Gastroenterology, American Association for the Study of Liver Diseases and European Association for the Study of the Liver guidelines owing to a lack of evidence on benefit and cost‐effectiveness [50, 51], in contrast, the American Association of Gastroenterology advises consideration for hepatocellular carcinoma in those with MASLD and advanced liver fibrosis [52]. Our findings suggest that HCC risk is higher than previously reported, and consideration should be given to screening for HCC in patients with MASLD and F3 fibrosis. Further work is underway to identify novel biomarkers for early detection of HCC in a large prospective cohort that may guide further economic evaluation of HCC screening in F3 MASLD [53].

The study was unable to meta‐analyse multivariable HRs reported in studies when considering MALO. Meta‐regression was attempted for sex, type 2 diabetes, and location and are available in the Supporting Information but should be interpreted with caution given so few studies are included. However, five studies provided univariable hazard ratios of MALO compared to control patients. F3 fibrosis had a pooled HR 8.15 (95% CI 3.42–19.43; *I* ^2^ 67.7%) in contrast; patients with F4 fibrosis developing MALO was over 4 times greater HR 38.16 (95% CI 11.58–125.76; *I* ^2^ 84.4%). While unsurprisingly, the risk of liver failure related events is higher in the patients with cirrhosis, the inflection point is not between the F3–F4 boundary but at the F2‐F3 boundary. We should perhaps consider not trying to force the available NITS beyond their capability to diagnose a histologically pure population of cirrhosis patients when the clinical complications of chronic liver disease are not confined to that group.

Our review has a number of strengths. First, a large number of patients were included across the papers, although we acknowledge that there is some overlap in the participants in the papers from paired biopsy trials. Second, we additionally sought papers that reported clinical outcomes making this review directly relevant to clinical practice and providing insight into the natural history of disease progression. The included studies also demonstrated a degree of ethnic diversity making conclusions applicable to clinical practice. There are limitations to this review which must also be acknowledged. First, there is significant heterogeneity in the data as noted in the meta‐analysis and when attempting to perform meta‐regression. Furthermore, the definitions of F3 and F4 fibrosis varied across included studies. Secondly, for those studies using liver biopsy to stage cirrhosis, the reporting of liver histology is known to have significant intra‐operator and inter‐operator variability, as well as sampling variability [54, 55], which may affect the interpretation of the results as the majority of included studies used this method for grading of fibrosis. The potential for publication bias must also be acknowledged, which we have attempted to minimise by including conference abstracts in the synthesis.

Our findings are comparable to other reports in the literature. An earlier review by Singh et al. found that there was 1 stage of fibrosis progression over 14.3 years in MASLD and 7.1 years in patients with MASH [15] but did not look at clinical outcomes. Another recent review looked at progression to cirrhosis in those with MASLD but did not break this down by baseline fibrosis stage [56]. They report risk of progression to cirrhosis in patients with simple steatosis is between 0% and 4%, with progression in MASH being reported in up to 10% in studies with up to 20 years of follow‐up. One study of paired biopsy samples in patients with MASLD found that 42% of patients had progression of fibrosis and 18% had regression of fibrosis over a median follow‐up period of 6.6 years [57]. The authors found that FIB‐4 score at the time of second biopsy and the presence of type 2 diabetes indicated possible fibrosis progression. Another study has suggested that those with worsening of their metabolic risk factors had progression of their fibrosis and development of MASH from MASLD [58].

Overall, our findings indicate that defining HCC and clinically significant portal hypertension (CSPH) as complications of histological stage F4 cirrhosis only is wrong and the risk of these complications is present in F3 stage. This may be due to the diagnostic limitations of liver biopsy under‐staging the disease or that the fibrosis of F3 is sufficient to start the pathophysiological processes of CSPH and HCC development. Non‐invasive tests that are effective at diagnosing a composite of F3–4 may, therefore, be of more use than liver biopsy.

## 5 Conclusions

To our knowledge, this is the first review looking specifically at the rate of liver disease complications in F3 fibrosis compared with F4 fibrosis in MASLD. We have illustrated that progression to cirrhosis or decompensation events occurs in a significant proportion of patients presenting with MASLD and F3 fibrosis. It takes roughly double the amount of time for patients with F3 fibrosis to develop decompensation events compared with those with F4 fibrosis. We found compelling evidence of the risk of developing HCC in F3 MASLD. These risks associated with F3 fibrosis must be high enough for intervention to be cost‐effective. It may be time to consider clinical outcome more important than histology and to consider patients with severe fibrosis to be at risk of the complications of chronic liver disease.

## Author Contributions

R.B.: methodology, investigation, data curation, writing – original draft, review and editing, visualisation. A.A.: formal analysis, writing – review and editing, visualisation. J.C.: methodology, investigation, writing – review and editing. K.A.: conceptualisation, methodology, formal analysis, writing – review and editing. J.F.D.: conceptualisation, methodology, writing – review and editing, supervision. P.N.B.: conceptualisation, methodology, writing – review and editing, supervision.

## Funding

The authors have nothing to report.

## Conflicts of Interest

R.B., J.C., A.A. and J.F.D. have no conflicts of interest to declare. K.A. has received consultancy fees from Novo Nordisk and lecturer honorarium from Advanz Pharma. P.N.B. discloses consultancy fees for Novo Nordisk and Resolution Therapeutics and educational honoraria from Takeda and Novo Nordisk outside of the submitted work.

## Supporting information


**Table S1:** Inclusion criteria.
**Table S2:** Pubmed search strategy.
**Table S3:** Scopus search strategy.
**Table S4:** Embase search strategy.
**Table S5:** Cochrane search strategy.
**Table S6:** Demographics of included studies.
**Table S7:** Univariable meta‐regressions.
**Table S8:** Event rates for development of decompensated liver disease or severe liver disease.
**Table S9:** Event rates for development of hepatocellular carcinoma.
**Figure S1:** Liver related events in F3 fibrosis meta‐analysis before and after greater RoB studies removed.
**Figure S2:** Liver related events in F4 fibrosis meta‐analysis before and after greater RoB studies removed.

## Data Availability

Further data are available at reasonable request.
