# Supplementary material for: Progression to Decompensation of Severe Fibrosis Compared to Cirrhosis in MASLD: A Systematic Review and Meta‐Analysis
Source: Liver Int. 2026 Jan 17;46(2):e70511. doi: 10.1111/liv.70511 (PMC12811796; doi:10.1111/liv.70511)
Supplement: Supplementary file 1 — Table S1: Inclusion criteria. Table S2: Pubmed search strategy. Table S3: Scopus search strategy. Table S4: Embase search strategy. Table S5: Cochrane search strategy. Table S6: Demographics of included studies. Table S7: Univariable meta‐regressions. Table S8: Event rates for development of decompensated liver disease or severe liver disease. Table S9: Event rates for development of hepatocellular carcinoma. Figure S1: Liver related events in F3 fibrosis meta‐analysis before and after greater RoB studies removed. Figure S2: Liver related events in F4 fibrosis meta‐analysis before and after greater RoB studies removed. [file LIV-46-0-s001.docx]

**Supplementary Table 1 Inclusion Criteria**

| **Criteria for Including Studies in the Review PICO question** | |
| --- | --- |
| Population or participants and conditions of interest | Adults of any age and gender with F3 liver fibrosis (NAFLD/NASH only/MAFLD/MASLD)  Keywords = “F3 liver fibrosis”, ”F3 fibrosis”, “bridging fibrosis”, “advanced fibrosis”, “fibrosis stag*”, “ prognos*”, “progression”, “predict”, ”natural history”, “fibrosis” |
| Interventions or exposures | Development of decompensation or progression to cirrhosis  In those with NAFLD/MAFLD/MASLD/NASH  Keywords = “NAFLD” “nonalcoholic fatty liver*” “nonalcoholic steatohepatit*” or “nafl*” OR “MASLD” or “metabolic dysfunction-associated steatotic liver disease” OR “NASH” |
| Comparisons or control groups | Adults with F4 fibrosis  Keywords = “F4 fibrosis”, “cirrhosis”, “cirrhosis complications”, “fibrosis”, “liver cirrhosis”, “severe liver disease” |
| Outcomes of interest | Decompensation  Keywords = “decompensat*”, “encephalopathy”, “variceal bleed*”, “varices”, “ascites”, “hepatocellular carcinoma”, “HCC” |
| Setting | Any setting |
| Study design | Any study design would be accepted but we are expecting cohort or case control studies rather than RCT |
| Diagnostic Modalities and Thresholds | Liver biopsy with metavir criteria:   - F0 no fibrosis can be detected - F1 portal fibrosis without septa - F2 portal fibrosis with rare septa - F3 numerous septa without cirrhosis - F4 cirrhosis(8)   Liver Stiffness Measurement VCTE (kPa), we will review definitions and rationale given by each paper, but cut offs should be in the region of:   - F2 >7.8kPa or 8.2kPa - F3 Fibrosis 9.7kPa - F4 fibrosis/cirrhosis > 16kPa or >13.6(kPa) (9,10)   Acoustic Radiation Force Impulse (ARFI):   - Per manufacturers for specific device   Magnetic Resonance Elastography (MRE) (kPa):   - F1 2.32 - F2 2.61 - F3 3.02 - F4 4.23(11)   We would also accept changes in fibrosis scores based on different modalities. |

**Search Strategies**

**Supplementary Table 2: Pubmed search strategy**

| Pubmed: 1322 results | |
| --- | --- |
| 1 | ("F3 liver fibrosis"[Title/Abstract] OR "F3 fibrosis"[Title/Abstract] OR "bridging fibrosis"[Title/Abstract] OR "advanced fibrosis"[Title/Abstract] OR "fibrosis stag*"[Title/Abstract] OR "prognos*"[Title/Abstract] OR "progression"[Title/Abstract] OR "predict*"[Title/Abstract] OR "Fibrosis"[Title/Abstract] OR "natural history"[Title/Abstract]) |
| 2 | ("F4 Liver Fibrosis"[All Fields] OR "F4 fibrosis"[Title/Abstract] OR "cirrhosis"[Title/Abstract] OR "cirrhosis complications"[Title/Abstract] OR "Fibrosis"[MeSH Terms] OR "Fibrosis"[Title/Abstract] OR "liver cirrhosis"[MeSH Terms] OR "severe liver disease"[Title/Abstract]) |
| 3 | ("decompensat*"[Title/Abstract] OR "encephalopathy"[Title/Abstract] OR "variceal bleed*"[Title/Abstract] OR "varices"[Title/Abstract] OR "ascites"[Title/Abstract] OR "hepatocellular carcinoma"[Title/Abstract] OR "HCC"[Title/Abstract]) |
| 4 | ("NAFLD"[Title/Abstract] OR “non-alcoholic fatty liver disease”[MeSH Terms] "nonalcoholic fatty liver*"[Title/Abstract] OR "nonalcoholic steatohepatit*"[Title/Abstract] OR "nafl*"[All Fields] OR "MASLD"[Title/Abstract] OR “metabolic dysfunction associated steatotic liver disease” [Title/Abstract] OR "NASH"[Title/Abstract]) |
| 5 | 1 AND 2 AND 3 AND 4 AND NOT "systematic review" [publication type] NOT "review" [publication type] |

**Supplementary Table 3: Scopus search strategy**

| Scopus 1439 results | |  |
| --- | --- | --- |
| 1 | (Title-Abs ({F3 liver fibrosis} OR {F3 fibrosis} OR {bridging fibrosis} OR {advanced fibrosis} OR {fibrosis stag*} OR {prognos*} OR {progression} OR {predict*} OR {Fibrosis} OR {natural history})) |  |
| 2 | (Title-Abs ({F4 Liver Fibrosis} OR {F4 fibrosis} OR {cirrhosis} OR {cirrhosis complications} OR {Fibrosis} OR {liver cirrhosis} OR {severe liver disease})) |  |
| 3 | (Title-Abs ({decompensat*} OR {encephalopathy} OR {variceal bleed*} OR {varices} OR {ascites} OR {hepatocellular carcinoma} OR {HCC})) |  |
| 4 | ((Title-Abs ({NAFLD} OR {non-alcoholic fatty liver disease} OR {nonalcoholic fatty liver*} OR {non-alcoholic steatohepatit*} OR {MASLD} OR {metabolic dysfunction associated steatotic liver disease} OR {NASH})) OR {nafl*}) |  |
| 5 | 1 AND 2 AND 3 AND 4 |  |

| Embase | | |
| --- | --- | --- |
| 1 | ("F3 liver fibrosis" or "F3 Fibrosis" or "bridging fibrosis" or "advanced fibrosis" or "fibrosis stag*" or "prognos*" or "progression" or "predict" or "Fibrosis" or "natural history").ab,ti. | 3016323 |
| 2 | ("F4 Liver Fibrosis" or "F4 Fibrosis" or "cirrhosis" or "cirrhosis complications" or "fibrosis" or "severe liver disease").ab,ti. | 503212 |
| 3 | exp liver cirrhosis/co, di, dm, ep, et, pc [Complication, Diagnosis, Disease Management, Epidemiology, Etiology, Prevention, Side Effect] | 32172 |
| 4 | exp liver fibrosis/co, di, dm, ep, et, pc, si [Complication, Diagnosis, Disease Management, Epidemiology, Etiology, Prevention, Side Effect] | 13608 |
| 5 | ("decompensate" or "encephalopathy" or "variceal bleed" or "varices" or "ascites" or "hepatocellular carcinoma" or "HCC").ab,ti. | 336123 |
| 6 | ("NAFLD" or "non-alcoholic fatty liver disease" or "non-alcoholic steatohepatit*" or "nafl*" or "MASLD" or "metabolic dysfunction associated steatotic liver disease" or "NASH").ab,ti. | 64051 |
| 7 | exp nonalcoholic fatty liver/co, di, dm, ep, et, pc, si, th [Complication, Diagnosis, Disease Management, Epidemiology, Etiology, Prevention, Side Effect, Therapy] | 13934 |
| 8 | 2 OR 3 OR 4 | 515218 |
| 9 | 6 OR 7 | 67385 |
| 10 | 1 AND 5 AND 8 AND 9 | 4642 |

**Supplementary Table 4: Embase search strategy**

**Supplementary Table 5: Cochrane Search Strategy**

| Cochrane | | |
| --- | --- | --- |
| 1 | ("F3 liver fibrosis" OR "F3 fibrosis" OR "bridging fibrosis" OR "advanced fibrosis" OR (fibrosis NEXT stag*) OR (prognos*) OR "progression" OR (predict*) OR "Fibrosis" OR "natural history") | 35536 |
| 2 | ("F4 Liver Fibrosis" OR "F4 fibrosis" OR "cirrhosis" OR "cirrhosis complications" OR "Fibrosis" OR "severe liver disease") | 8149 |
| 3 | "Fibrosis"[MeSH Terms] OR "liver cirrhosis"[MeSH Terms] | 4392 |
| 4 | 2 OR 3 | 10607 |
| 5 | (decompensat*) OR "encephalopathy" OR (variceal NEXT bleed*) OR "varices" OR "ascites" OR "hepatocellular carcinoma" OR "HCC” | 7321 |
| 6 | ("NAFLD"OR (nonalcoholic NEXT fatty NEXT liver*) OR (nonalcoholic NEXT steatohepatit*) OR (nafl*) OR "MASLD" OR “metabolic dysfunction associated steatotic liver disease” OR "NASH" | 3158 |
| 7 | “non-alcoholic fatty liver disease”[MeSH Terms] | 1634 |
| 8 | 6 OR 7 | 3587 |
| 9 | 1 AND 4 AND 5 AND 8 | 1 |

**Supplementary Table 6: Demographics of Included Studies**

| **Author** | **Year** | **Country** | **Number of Participants** | **Age** | **Sex** | **Ethnicity** | **BMI** | **Co-morbidity** |
| --- | --- | --- | --- | --- | --- | --- | --- | --- |
| Younossi | 2021 | USA, UK, Austria | 2154 | Mean: 57.1 years  SD 8.9) | Male: 40% | N/A | Mean: 33.7 (SD 6.6) | Type 2DM: 72% |
| Chan | 2014 | Malaysia | 35 paired biopsies | Worsened NAS: 44.1 years  Improved or unchanged NAS: 50.5 years | Worsened NAS male: 46.2%  Improved or unchanged NAS male: 36.4% | Worsened NAS:  Malay 9(69.2%)  Chinese 3 (23.1%)  Indian 1 97.7%)  Unchanged or improved NAS:  Malay 10 (45.4%)  Chinese 6 (27.3%)  Indian 6 (27.3%) | Worsened NAS: mean 25.1 (SD 3.6)  Improved or unchanged NAS: mean 29.2 (SD 4.6) | Hyperlipidaemia: 97.1%  Diabetes Mellitus: 54.3% Hypertension: 31.4% |
| Huang | 2023 | USA | 448 | Mean: 50.9 years (SD 11.5) | Male: 163 (36%)  Female: 284 (64%) | White: 381 (85%)  Other or not reported: 66 (15%)  Hispanic ethnic group:  Yes 46 (10%)  No 401 (90%) | Mean: 34.7 (SD 6.3) | Hypertension: 256 (57%)  Coronary artery disease: 19 (4%)  Metabolic syndrome: 298 (67%) |
| Lin | 2019 | Taiwan | 10 | Mean: 44 years (range 28-65 years) | Male: 7 (70%) | Taiwanese | Obesity: 4 (40%) | Metabolic Syndrome: 7 (70%) |
| Loomba | 2023 | USA/Multi-national | F3 fibrosis: 664  F4 fibrosis: 734 | Median (IQR)  F3: 58 years (51-54)  F4: 59 years (53-65) | F3: 56% female  F4: 63% female | F3:  72% white  14 % Hispanic/Latino  F4:  78% white, 13% Hispanic/Latino | Median (IQR)  F3: 32.7 (29-37)  F4: 33.1 (28.7-38) | Diabetes:  F3 446/664 (70%)  F4: 556/734 (76%) |
| Kleiner | 2019 | USA | 446 | Mean (SD)  NAFLD: 45 years (11)  Borderline steatohepatitis: 45 years (11)  Definite steatohepatitis: 49 years (11) | Male  NAFLD: 34 (39.5%)  Borderline steatohepatitis: 34 (40.5%)  Definite steatohepaitis: 84 (30.4%) | White  NAFL: 74 (89.2%)  Borderline steatohepatitis: 71 (89.9%)  Definite steatohepatitis: 229 (87.1%) | Mean (SD)  NAFL: 34.1 (7.1)  Borderline Steatohepatitis: 34.5 (5.3)  Definite Steatohepaitits: 35.9 (6.9) | Diabetes  NAFL: 18 (20.9%), Borderline steatohepatitis: 16 (19.0%)  Definite steatohepatitis: 115 (41.7%) |
| Kobayashi | 2023 | Japan | 405 | Mean 58.5 years (SD 2.5) | Female: 219 (54.1%) | All Japanese | Mean (SD)  29.9 (25.9) | Diabetes 217 (53.5%)  HTN 218 (53.7%) |
| Hagstrom | 2017 | Sweden | 646 patients with liver biopsy and NAFLD | Mean: 48.2 years (SD 13.7) | Male 62.2% | N/A | Mean (SD)  28.3 (4.1) | HTN: 3.03%  T2DM: 14.4% Smoking: 24% Previous smoking: 21% |
| Kodama | 2019 | Japan | 104 patients with NAFLD-HCC | Median (range)  F0-F2: 71 years 41-86)  F3-F4: 70 years (45-89) | Female/Male: 7/28 in F0-2  Female/Male: 29/40 in F3-4 | N/A | F0-2: 25.4  F3-4: 27.4 | T2DM  F0-2: 71%  F3-4: 68%  HTN  F0-F2: 66%, F3-4: 62%  Dyslipidaemia  F0-2: 62%, F3-F4: 36% |
| Nakamura | 2008 | Japan | 72 | Median: 62 years (range 16-89) | Male: 35  Female: 37 | N/A | Obesity defined as BMI > 25 kg/m2: 56 (77.8%) | N/A |
| Gadi | 2023 | Belgium | 100 | Progressed to decompensation: 64.8 years  Did not progress to decompensation: 58.1 years | N/A | N/A | N/A | N/A |
| Gawrieh | 2022 | USA | 894 | N/A | N/A | Progressors versus non-progressors:  White: 90% vs 80%  Non-Hispanic 95% vs 88% | Progressors versus non-progressions:  34 vs 32 | Progressors versus non-progressor:  Obesity: 7s9% vs 61% |
| Kawada | 2009 | Japan | 807  8 with NASH HCC  6 with non-cirrhotic NASH HCC | Median 73 years | n=6  Male: 3/6 (50%)  Female: 3/6 (50%) | N/A | n=6  Median 23.6 (range 21.8-28.1)  Obesity: 2 (33%) | n=6  Diabetes: 3/6  HTN: 4/6  Hyperlipidaemia: 4/6 |
| Mohamad | 2015 | USA | 83 | Mean (SD)  64.8 years (10.4) | Male: 54 (65.1%) | White: 92.8% African American: 4.8% | BMI > 30: 69.% | Diabetes: 63.9%  Metabolic syndrome: 84% HTN: 77.9% |
| Ekstedt | 2006 | Sweden | 88 | Mean (SD)  51.0 years (12.9) | Male: 87 (67%) | N/A | Mean (SD)  28.3 +/- 3.8  Obese: 37 (29%) | Diabetes at baseline: 11 (8.5%)  HTN: 93(72%)  Cardiovascular disease: 14 (11%)  Hypertriglyceridemia: 74 (57%) |
| Duseja | 2022 | India | 4313  3553 with full data on metabolic risk factors | Mean (SD)  45.2 years (12.2) | M: 2249 (52.1%)  F: 2064 (47.9%) | N/A | BMI:  < 18: 16 (0.45%)  18-22.99: 378 (10.6%)  23-24.99: 575 (16.2%)  >=25: 2584 (73) | HTN: 15.5%  Diabetes Mellitus: 21.9% |
| Fujii | 2023 | Japan | 1398 | Mean (SD)  54.5 years (14.2) | Male: 599 (45.8%) | N/A | BMI: 28 (4.7) (n=1395) | DM, HTN, Dyslipidaemia |
| Hirose | 2018 | Japan | 233 | N/A | N/A | N/A | N/A | NASH patients: higher prevalence of diabetes (25.4% versus 7%) |
| Lam | 2022 | USA | 450 | Median: 52 years | Male: 43% | N/A | Obesity (67%) | HTN (58%)  Diabetes (47%)  Coronary artery disease (9%) |
| Vilar-Gomez | 2018 | USA | 458 | Mean (SD)  F3: 54.2 years ( 10.7)  F4 CTP A5: 56.6 years (11.8)  F4 CTP A6: 57.4 years (10.4) | F3: male 79 (50%)  F4 CTP A5: 99 (46%)  F4 CTP A6: 40 (52%) | F3  Hispanic white: 76 (48%)  Non-Hispanic white: 45 (28%)  Asian: 28 (24%)  Black: 0  F4 CTP A5  Hispanic white: 123 (55%)  Non-Hispanic white: 49 (22%)  Asian: 46 (21%)  Black: 4 (2%)  F4 CTP A6:  Hispanic white: 57 (74%)  Non-Hispanic white: 18 (23)  Asian: 2 (3%)  Black 0 (0) | BMI 33.2 +/- 8.6 | In all groups  Former smoking: 59 (13%)  Current smoking: 78 (17%)  Alcohol consumption:  non-drinkers 392 (86%), moderate drinkers 66 (14%)  HTN 281 (61%)  T2DM 305 (67%) |
| Angulo | 2015 | USA/Mutli-national | 619 | Median (IQR)  49 years (38-60) | Female: 387 (62.5%)  Male: 232 (37.5%) | Ethnicity  Hispanic: 1 (0.2%), Non-Hispanic: 618 (99.8%)  Race  White: 545 (88%), Asian: 45 (7.3%), Black or African American: 1 (0.2%), More than 1 race: 28 (4.5%) | Median (IQR)  30.7 (26.4-36.5) | Diabetes: 232 (37.5%)  HTN: 190 (30.7%)  Statin: 63 (10.2%)  Ex-smoker: 72 (11.6%)  Current smoker: 54 (8.7%) |
| Sanyal | 2021 | USA | 1773 | Mean 52 years | Female: 64% Male: 36% | White: 1509 (85%)  Other or not reported: 264 (15%)  Hispanic ethnic group: 217 (12%) | Median (IQR)  F4: 35 (31-40)  F3: 34 (31-40)  F0-2: 33 (29-37) | HTN: 1073 (61%)  Diabetes: 742 (42%)  CKD: 99 (6%)  Previous non-hepatic primary cancer 181 (10%) |
| Sanyal | 2019 | USA | 217: bridging fibrosis  258: compensated cirrhosis | Median (IQR)  Bridging Fibrosis:  55 years (48-59)  Compensated cirrhosis:  57 years (51-61) | Bridging Fibrosis: female 137 (63%)  Cirrhosis: 163 (63%) | Bridging Fibrosis  White: 205 (94%)  Hispanic/Latino: 34 (16%)  Cirrhosis  White: 238 (92%)  Hispanic/Latino: 39 (15%) | Median (IQR)  Bridging fibrosis: median: 33.7 (30.3-38.4)  Cirrhosis: 33.6 (29.7-38.2) | Diabetes:  Bridging fibrosis: 145 (67%)  Cirrhosis: 179 (69%) |
| Bengtsson | 2018 | Sweden | 1562 | 72 years in NAFLD-HCC group | NAFLD group: male sex 70.7% | N/A | N/A | NAFLD group  HTN: 75.5%  T2DM: 74.7%  CV disease: 29.8%  Hyperlipidaemia: 28.6% |
| Yasui | 2011 | Japan | 87 | Median (IQR)  72 years (69-75) | Male: 54 (63%)  Female: 33 (38%) | N/A | Median: 26  Obese (BMI> 25): 54 (62%) | Diabetes: 51(59%)  Dyslipidaemia: 24 (28%)  HTN: 47 (55%) |
| Pinyopornpanish | 2022 | USA | 2237 patients (total)  NAFLD 346 | Mean (SD)  63.3 years (10.3) | Male 78.5% | Caucasian: 78.8%  African-American: 13.8%  Asian: 3.2%  Hispanic: 1.8% | Mean 28.5 (SD 5.9)  (all HCC patients not just NAFLD aetiology) | HTN: 1293 (57.8%)  Diabetes mellitus: 814 (36.4%)  Hyperlipidaemia: 569 (26.2%)  Ever smoker: 1614 (72.4%)  (all patients not just NAFLD aetiology) |
| Davitkov | 2023 | USA | 13629 with NAFLD  42 of these had HCC | LSM < 12.5kPa: 56.2 years  LSM >12.5kPa: 61.9 years | LSM < 12.5kPa:  Male 89%  LSM >12.5kPa:  Male 93 % | White  LSM < 12.5kPa: 69%  LSM >12.5kPa: 79%  Black  LSM < 12.5kPa: 20%  LSM >12.5kPa: 12%  Hispanic  LSM < 12.5kPa: 10%  LSM >12.5kPa: 10% | Median (SD)  LSM < 12.5kPa: 32.6 (5.4)  LSM >12.5kPa: 34.8 (6.6) | Alcohol  LSM < 12.5kPa: 1191 (11%)  LSM > 12.5kPa: 374 (14%)  Tobacco  LSM < 12.5kPa: 622 (6%)  LSM > 12.5kPa: 176 (7%)  Diabetes  LSM < 12.5kPa: 3886 (35%)  LSM > 12.5kPa: 1708 (64%) |
| Campos | 2017 | Brazil | 21 | Range: 50-77 years | Male: 16 (76%) | N/A | BMI > 25: 19 patients (90%) | Diabetes Mellitus:  16 patients (76%)  Hypertension: 17 patients (81%) |
| Wong | 2010 | Hong Kong | 52 | Mean (SD)  44 years (9) | Male 34 (65%) | N/A | Mean (SD)  Male: 27.7 (4.1) Female: 27.0 (2.9) Overall 27.4 (3.7) | Diabetes: 26 (50%)  Metabolic Syndrome: 35 (67%) |

**Sensitivity Analysis**

We have performed a sensitivity analysis excluding the studies rated ‘fair’ quality by the risk of bias analysis – these were *Angulo et al* and *Hirose et al*. The sensitivity analysis demonstrates that removing the fair studies has little impact on the pooled hazard ratios effect estimate but does significantly reduce precision, with much wider confidence intervals.


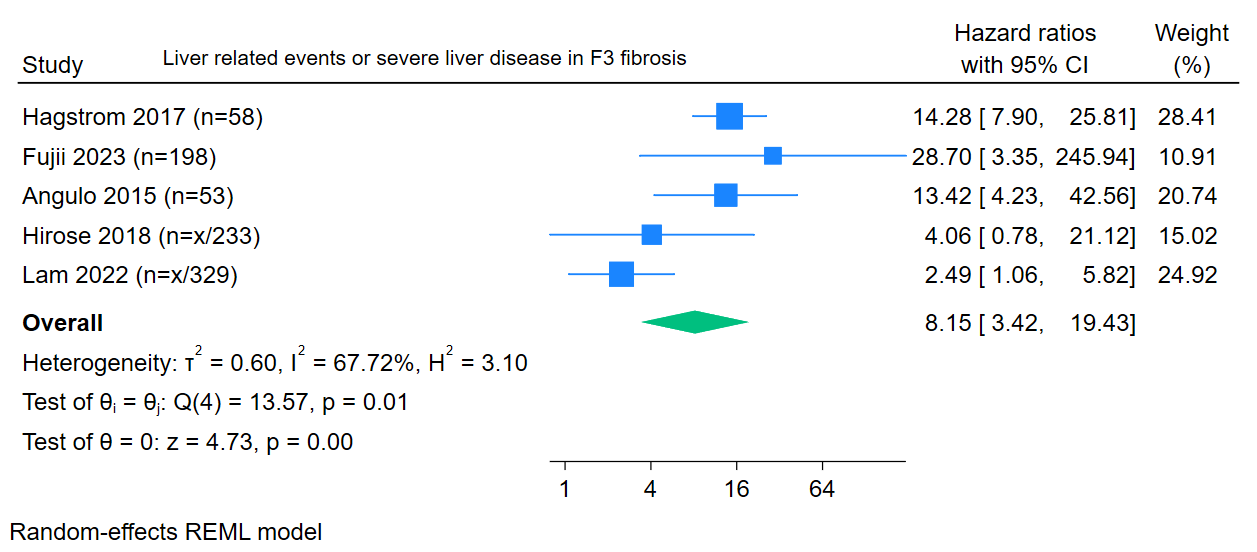


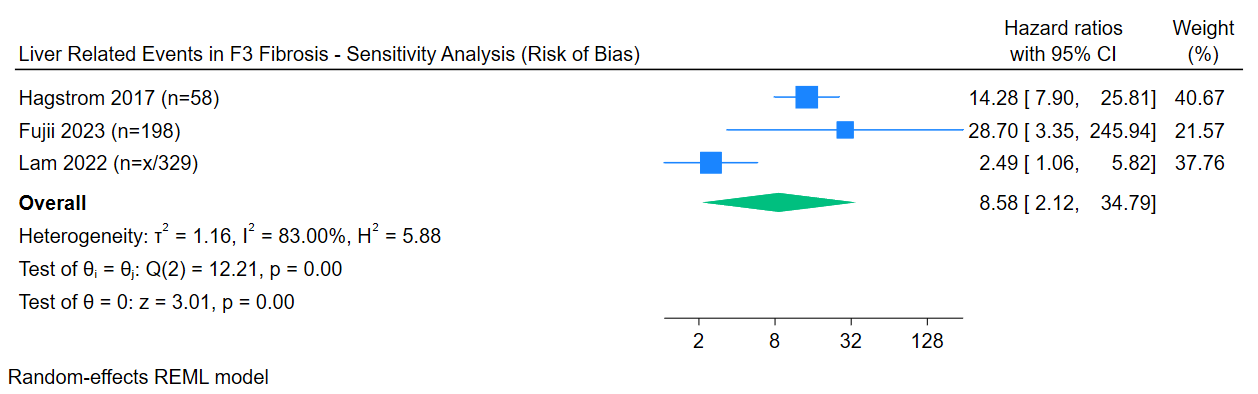


**Supplementary Figure 1** – Liver related events in F3 fibrosis meta-analysis before and after greater RoB studies removed


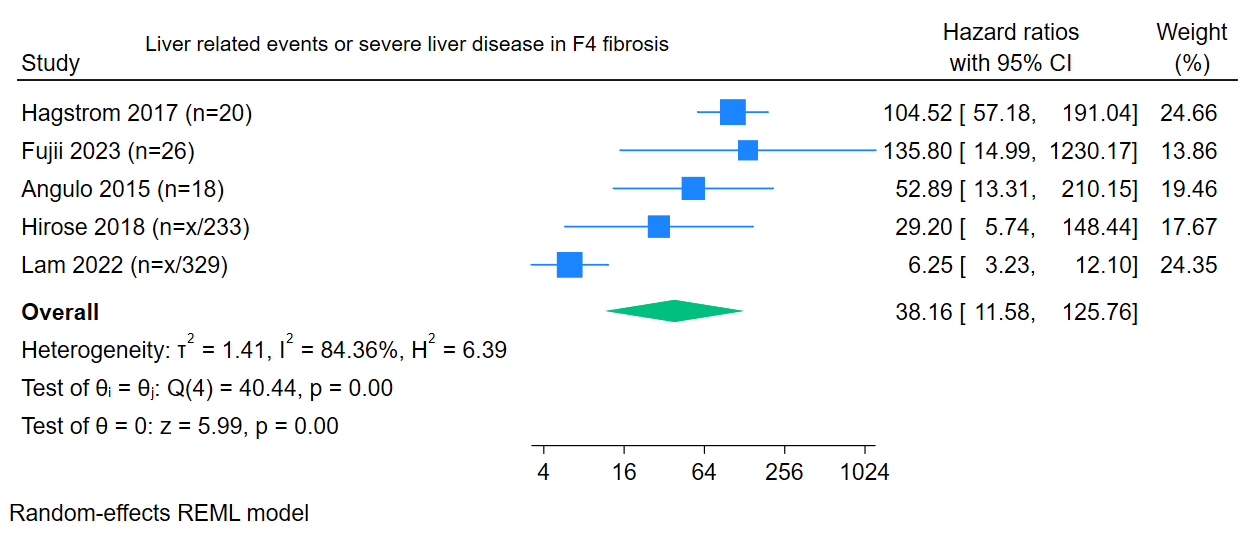


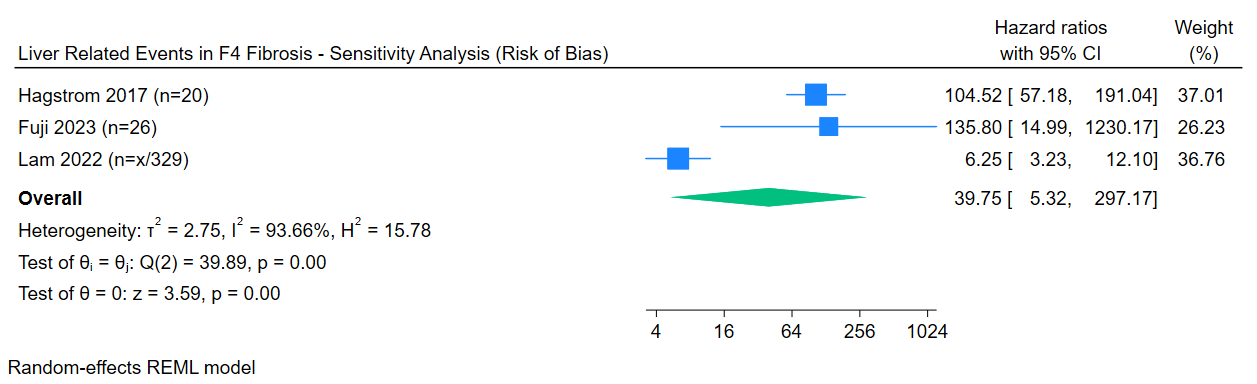


**Supplementary Figure 2** – Liver related events in F4 fibrosis meta-analysis before and after greater RoB studies removed

| **Liver related events or severe liver disease in F3 fibrosis meta regression** | | | | | |
| --- | --- | --- | --- | --- | --- |
| **Moderator** | **β coefficient (95% CI)** | **p-value** | **τ²** | **I² (%)** | **k** |
| Sex (% male) | 0.240 (−0.101 to 0.149) | 0.706 | 1.205 | 75.73 | 4 |
| T2DM (%) | -0.132 (-0.074 to 0.048) | 0.672 | 0.746 | 65.41 | 5 |
| Location (Western) | -0.191 (-2.357 to 1.974) | 0.862 | 0.833 | 76.97 | 5 |
|  |  |  |  |  |  |
| **Liver related events or severe liver disease in F4 fibrosis meta regression** | | | | | |
| **Moderator** | **β coefficient (95% CI)** | **p-value** | **τ²** | **I² (%)** | **k** |
| Sex (% male) | 0.058 (-0.109 to 0.224) | 0.497 | 2.233 | 84.96 | 4 |
| T2DM (%) | -0.038 (-0.110 to 0.034) | 0.299 | 1.176 | 73.20 | 5 |
| Location (Western) | -0.603 (-3.384 to 2.179) | 0.671 | 1.762 | 88.71 | 5 |

**Supplementary Table 7: Univariable meta-regressions**

**Supplementary Table 8**

| **Event rate (per 1000 person years) for development of decompensated liver disease or severe liver disease** | | | | |
| --- | --- | --- | --- | --- |
| Decompensated Liver Disease | | | | |
| **Study** | **F3 Fibrosis** | **F4 Fibrosis** | **Follow-up period (1000 person- years)** | **Definition in Paper** |
| Sanyal 2021 | 2.1 | 2.1 | 8.12 | New onset hepatic decompensation = clinically apparent ascites, overt encephalopathy or variceal haemorrhage |
| Ekstedt | 2.5 | 0.8 | 1.20 | End-stage liver disease = chronic hepatic failure, portal HTN, hepatorenal syndrome, oesophageal varices, HCC |
| Liver Related Event or Severe Liver Disease | | | | |
| **Study** | **F3 Fibrosis** | **F4 Fibrosis** | **Follow-up period (person- years)** | **Definition in Paper** |
| Hagstrom | 1.9 | 1.9 | 12.63 | Severe Liver Disease = ICD code for liver failure, cirrhosis, HCC or decompensated liver disease  Decompensated liver disease in turn defined as ICD-code: oesophageal varices (bleeding or not bleeding), ascites or hepatic encephalopathy. |
| Angulo | 0.9 | 0.5 | 7.80 | Liver related event: GOV or bleeding, ascites, portosystemic encephalopathy, SBP, HCC hepatopulmonary syndrome, hepatorenal syndrome |
| Vilar-Gomez | 5.7 | 30.4 (CTP A5)  108.4 (CTP A6) | 1.41 | Hepatic decompensation = first occurrence of ascites, upper gastrointestinal bleeding secondary to portal hypertension, or hepatic encephalopathy |

**Supplementary Table 9**

| **Event rate (per 100 person years) for development of Hepatocellular Carcinoma** | | | |
| --- | --- | --- | --- |
| **Study** | **F3 Fibrosis** | **F4 Fibrosis** | **Follow-up period (person- years)** |
| Sanyal 2021 | 0.7 | 0.1 | 8120 |
| Vilar-Gomez | 2.3 | 9.9 (CTPA5)  19.7 (CTPA6) | 880/1216/406 |


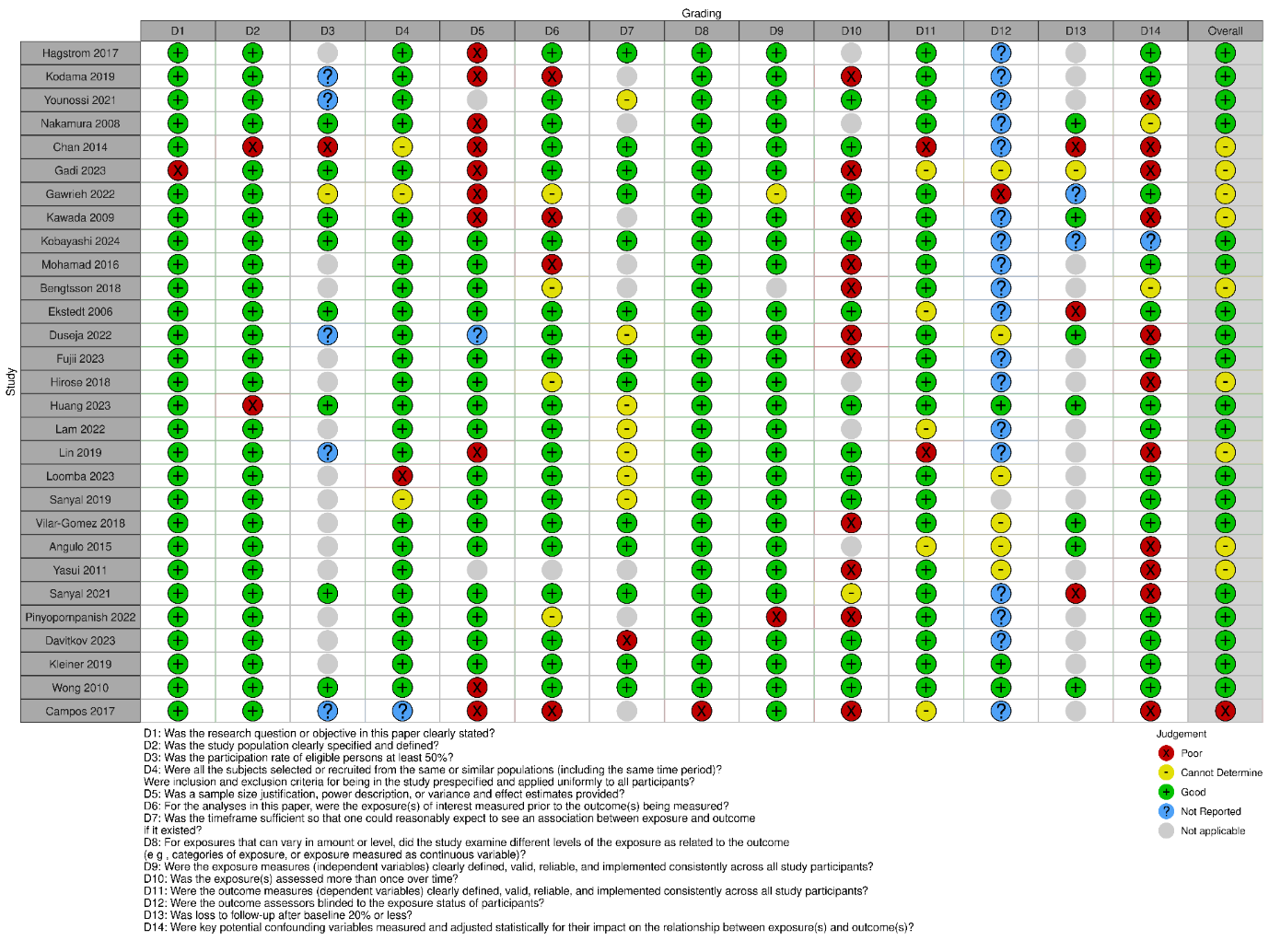
**Assessment of Bias ROBVIS Plot**

ROBVIS plot for assessment of bias. * in the “overall” column, “cannot determine” refers to a judgement of “fair”
